# A Two-Clone Approach to Study Signaling Interactions among Neuronal Cells in a Pre-clinical Alzheimer's Disease Model

**DOI:** 10.1016/j.isci.2020.101823

**Published:** 2020-11-18

**Authors:** Catherine J. Yeates, Ankita Sarkar, Prajakta Deshpande, Madhuri Kango-Singh, Amit Singh

**Affiliations:** 1 Department of Biology, University of Dayton, Dayton, OH 45469, USA; 2 Premedical Program, University of Dayton, Dayton, OH 45469, USA; 3 Center for Tissue Regeneration and Engineering at Dayton (TREND), University of Dayton, Dayton, OH 45469, USA; 4 The Integrative Science and Engineering Center, University of Dayton, Dayton, OH 45469, USA; 5 Center for Genomic Advocacy (TCGA), Indiana State University, Terre Haute, IN, USA

**Keywords:** Cell Biology, Molecular Biology, Neuroscience

## Abstract

To understand the progression of Alzheimer's disease, studies often rely on ectopic expression of amyloid-beta 42 (Aβ42) throughout an entire tissue. Uniform ectopic expression of Aβ42 may obscure cell-cell interactions that contribute to the progression of the disease. We developed a two-clone system to study the signaling cross talk between GFP-labeled clones of Aβ42-expressing neurons and wild-type neurons simultaneously generated from the same progenitor cell by a single recombination event. Surprisingly, wild-type clones are reduced in size as compared with Aβ42-producing clones. We found that wild-type cells are eliminated by the induction of cell death. Furthermore, aberrant activation of c-Jun-N-terminal kinase (JNK) signaling in Aβ42-expressing neurons sensitizes neighboring wild-type cells to undergo progressive neurodegeneration. Blocking JNK signaling in Aβ42-producing clones restores the size of wild-type clones.

## Highlights

• In the two-clone system a subset of neurons in a field expresses high levels of Aβ42
• A genetic recombination event generates Aβ42-expressing (GFP +ve) and WT neurons
• Surprisingly, WT neurons die prior to widespread death of Aβ42-expressing neurons
• Higher levels of JNK signaling in Aβ42-expressing cells causes death of WT neurons

## Graphical Abstract

**Figure:**
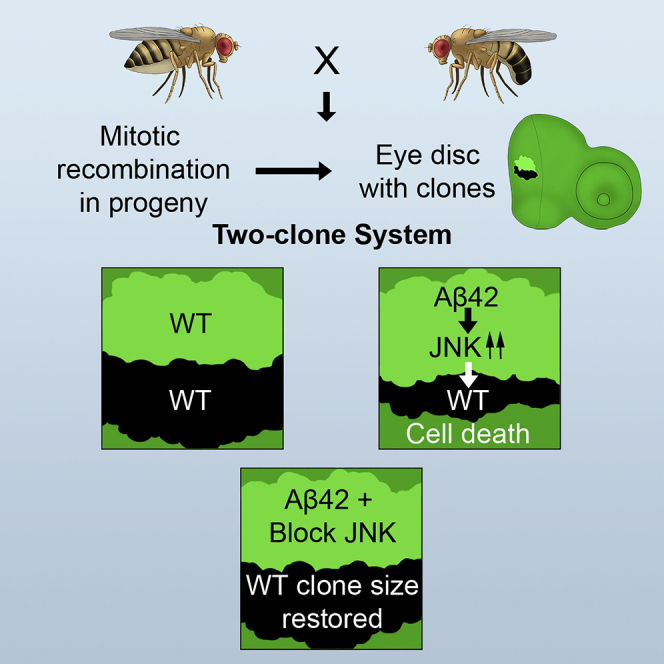

## Introduction

Alzheimer's disease (AD) is a debilitating neurodegenerative disorder that is marked by widespread cell death throughout the brain and progressive impairments to memory and cognitive function (McKhann et al., 1984). One of the hallmarks of AD is the accumulation of amyloid beta (Aβ) peptides in extracellular plaques (Bonini and Fortini, 2003; Cline et al., 2018; Glenner and Wong, 1984; Hardy, 2009; Tare et al., 2011). These extracellular plaques are accompanied by the aggregation of intracellular neurofibrillary tangles (NFTs) made up of hyperphosphorylated tau protein (Grundke-Iqbal et al., 1986; Kosik et al., 1986; Wood et al., 1986). The neuropathology of AD results in accumulation of Aβ42 plaques and NFTs, which triggers progressive neurodegeneration across brain regions (Braak and Braak, 1991; Palmqvist et al., 2017). It is not well understood how cellular changes contribute to the progression from an initial asymptomatic period into a phase of stark cognitive decline (Sperling et al., 2014).

The etiology of AD includes a complicated interplay between the accumulation of Aβ42 and hyperphosphorylated tau and other pathological changes including alteration of calcium regulation, dysfunction of mitochondria, and dysregulation of glia (Cline et al., 2018; Hansen et al., 2018). The brain is not uniformly affected by the disease, and it is not well understood how interactions between neurons affected by disease pathology and healthy neurons might contribute to the progression of AD over time (Goldman et al., 2018; Yeates et al., 2019). One possible point of failure in translation could be the difficulty in accurately modeling the local cellular context of the disease.

Transgenic models that express Aβ42 uniformly throughout entire tissues—such as the brain or retinal neurons—do not necessarily recapitulate the spread of AD pathology throughout the human brain (Fernandez-Funez et al., 2013; Jankowsky and Zheng, 2017; Sarkar et al., 2016). Changes to cell-cell signaling downstream of Aβ42 accumulation can result in aberrant activation of cell death pathways (Casas-Tinto et al., 2011; Gogia et al., 2021; Tare et al., 2011; Yarza et al., 2015; Yeates et al., 2019).The evidence for substantial dysregulation of cell death pathways in AD suggests that there is much more to be learned about local cellular changes that precede cell death as well as what predisposes certain cell populations to die. However, the signaling interactions between cells producing Aβ42 and neighboring cells are difficult to model in transgenic animals that ectopically express human Aβ42 uniformly using neuronal promoters (Drummond and Wisniewski, 2017).

*Drosophila melanogaster* is a versatile model organism and shares substantial conservation of basic genetic machinery and disease-related genes with humans (Bier, 2005). Its short life cycle and array of genetic tools make it a good organism for studying neurodegenerative disease (Deshpande et al., 2019; Gogia et al., 2020b, 2021; Iijima-Ando and Iijima, 2010; McGurk et al., 2015; Sarkar et al., 2016; Singh, 2012; Singh and Irvine, 2012). Adult flies possess a compound eye comprising around 800 individual units called ommatidia, which include retinal neurons and accessory cells (Gogia et al., 2020a; Ready et al., 1976; Singh et al., 2005, 2012; Tare et al., 2013a; Treisman, 2013). The eye develops from the eye-antennal imaginal disc in the larva, a monolayer epithelium that contains the differentiating retinal neurons (Kango-Singh et al., 2003; Raj et al., 2020; Ready et al., 1976; Singh and Choi, 2003; Singh et al., 2002, 2005).

Fly models of AD can successfully recapitulate elements of disease pathology such as amyloid plaque aggregation, cell death, and defects in learning and memory. Since the eye is not required for viability or fertility, severe neurodegenerative phenotypes can be studied. Human Aβ42 polypeptides can be misexpressed in the developing retinal neurons of transgenic fruit flies using the Gal4-UAS target system (Cao et al., 2008; Cutler et al., 2015; Moran et al., 2013; Steffensmeier et al., 2013; Tare et al., 2011). The enhancer for Glass Multiple Repeat (GMR) has been used to drive expression in the developing retina of *Drosophila* larvae (Brand and Perrimon, 1993; Moses and Rubin, 1991). GMR-Gal4 can be used to drive expression of human Aβ42 tagged with a signal peptide to trigger its extracellular transport in the differentiating retinal neurons of the developing retina. These flies develop extracellular Aβ42 plaques and progressive neurodegeneration through the larval, pupal, and adult stages. When Aβ42 is expressed in the entire eye field of third-instar larvae, their eye-antennal imaginal discs show disorganization and gaps in the spacing of retinal neurons (Tare et al., 2011). These neurodegenerative phenotypes are accompanied by an increase in cell death markers and in reporters that show upregulation of c-Jun N-terminal kinase (JNK) signaling (Sarkar et al., 2018; Singh et al., 2006; Tare et al., 2011).

In humans and AD models, Aβ42 accumulation is linked to increases in activation of the JNK pathway (Irwin et al., 2020; Ray et al., 2017; Sarkar et al., 2018; Tare et al., 2011; Wang et al., 2014; Yarza et al., 2015). The JNK signaling pathway, part of the mitogen-activated protein kinase (MAPK) superfamily, is highly conserved and transcriptionally activates apoptosis. Initiation of the pathway begins with Eiger, the fly homolog of the tumor necrosis factor (TNF), binding to Wengen and Grindelwald, the TNF receptors (Igaki et al., 2002; Kanda et al., 2002; Moreno et al., 2002). Activation of the TNF receptors allows the signal to be transmitted by *hemipterous* (*hep*), the *Drosophila* homolog of the Jun kinase kinase (JNKK) and a core component of this pathway (Glise et al., 1995; Sluss et al., 1996; Tournier et al., 1997). Basket (*bsk*), the fly JNK, is activated by phosphorylation, and in turn, it phosphorylates and activates the transcription factor *Drosophila* Jun related antigen (Jra or dJun) (Sluss et al., 1996). Puckered, a dual specificity phosphatase, is a transcriptional target of JNK signaling and regulates JNK signaling through a negative feedback loop (Adachi-Yamada et al., 1999; Adachi-Yamada and O'Connor, 2002; Martín-Blanco et al., 1998). JNK signaling triggers cell death both through activation of caspases *reaper* (*rpr*) and *head involution defective* (*hid*) and through caspase-independent mechanisms (Martín-Blanco et al., 1998; Singh et al., 2006).

Research in both flies and humans has implicated activation of the JNK signaling pathway in AD and other neurodegenerative disorders (Gogia et al., 2020b; Irwin et al., 2020; Tare et al., 2011; Wang et al., 2014; Yarza et al., 2015). JNK signaling has also been connected to a conserved process known as cell competition, which is involved in maintaining tissue homeostasis. Cell-cell signaling can occur both through secretion of signals and through expression of cell surface markers. Differential expression of cell surface proteins determines a cell's fitness relative to its neighboring cells, and less fit cells may be targeted for cell death in order to maintain tissue integrity (Coelho and Moreno, 2019). Previous research has tied cell competition to AD and further implicated the JNK pathway in the apoptotic cell death that can occur during cell competition (Casas-Tintó et al., 2015; Coelho et al., 2018; Ryoo et al., 2004).

In order to understand how changes in cell-cell signaling downstream of Aβ42 accumulation contribute to the progressive neurodegeneration seen in AD, we have developed a two-clone approach. Here we present a genetically tractable system to uncover new insights into interactions between labeled (GFP-positive) clones of Aβ42-expressing neurons and GFP-negative wild-type (WT) sister clones in the same tissue. In this system, we can model the onset of AD pathology and assess early neurodegeneration by triggering Aβ42 expression in the retina. The eye is not required for viability (Tare et al., 2013b), and this clonal analysis allows us to express Aβ42 in only a random subset of retinal cells. We can then assess neurodegeneration and changes to cell-cell signaling in these clones.

## Results

### Expression of Aβ42 in Neuronal Clones Leads to a Reduction in the Size of WT Sister Clones

**Figure 1:**
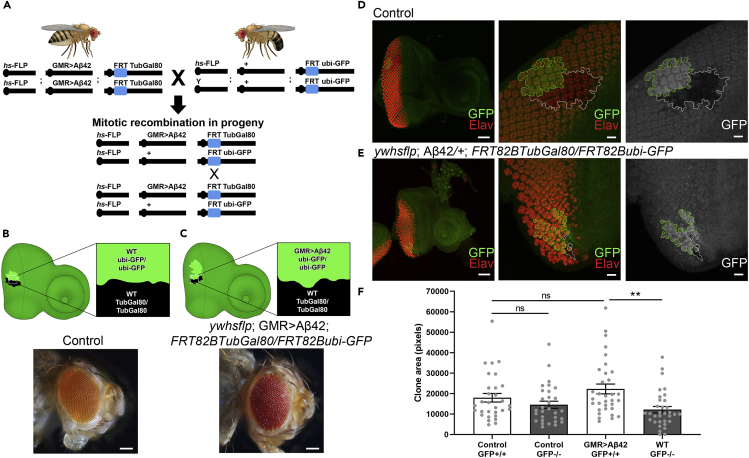
Presence of Aβ42-Expressing Clones Leads to a Preferential Decrease in Size of WT Sister Clones (A) Diagram of the crossing scheme and mitotic recombination generating Aβ42-expressing clones and WT sister clones. Parental lines were crossed to generate progeny of the genotype *y w hsflp; GMR > Aβ42/+; FRT82BTubGal80/FRT82Bubi-GFP*. A heat shock was applied to trigger mitotic recombination at the FRT sites, resulting in two clones of cells. (B) In a control background, GFP-positive (ubi-GFP/ubi-GFP; hereafter Control GFP+/+) and GFP-negative (TubGal80/TubGal80; hereafter Control GFP−/−) sister clones were generated. Both populations comprise WT cells. Eyes of adult flies with WT clones are normal in size and appearance. (C) As diagrammed in (A), mitotic recombination results in eye discs with two populations: one clone is GFP positive and expresses Aβ42 (ubi-GFP/ubi-GFP; hereafter GMR > Aβ42 GFP+/+) and the sister clone is GFP negative and WT, owing to the presence of two copies of the repressor TubGal80/TubGal80 (hereafter WT GFP−/−). In animals with Aβ42-expressing clones, eyes of adults are normal sized but show areas of roughness and irregular structure indicative of neurodegeneration. Scale bars in (B) and (C), 100 μm. (D) Control eye-antennal imaginal disc with GFP-positive and GFP-negative WT sister clones. Elav (red) marks the developing retinal neurons. Scale bars for 20× images, 50 μm. The following panels correspond to the same eye disc imaged at 40×, clones outlined. Elav staining shows that the retinal neurons of controls are regularly spaced and arranged. GFP-positive WT and GFP-negative WT clones are similar in size. (E) GFP-positive clones express Aβ42, and GFP-negative clones are WT. Elav staining shows gaps in spacing and disorganization indicative of the loss of retinal neurons. The WT clone is substantially reduced in size compared with the Aβ42-expressing clone. Scale bars for 40× images, 10 μm. (F) Clone sizes were quantified and compared. Statistical analysis was done using one-way ANOVA with Tukey's post hoc to compare these four groups. Data are presented as mean ± SEM. Control GFP-positive WT and GFP-negative sister clones were not significantly different (N = 30, p = 0.63), whereas WT clones adjacent to Aβ42-expressing sister clones were significantly smaller (N = 33, ∗∗p < 0.01). See also Table S1.

We have employed a genetic mosaic approach in which labeled clones of neurons are produced in the developing retina of *Drosophila melanogaster* larvae. We have used the FLP/FRT system in combination with the Gal4/UAS/Gal80 system. The FLP/FRT system triggers mitotic recombination mediated by Flippase (FLP) at Flippase Recognition Target (FRT) sites while the Gal4/UAS/Gal80 tissue-specific expression system can be used to introduce targeted misexpression of genes of interest, such as human Aβ42 (Figure 1A) (Lee and Luo, 1999; Xu and Rubin, 1993). Heterozygous larvae possess one copy of ubi-GFP (GFP under a ubiquitin promoter) and one copy of the Gal4 repressor, TubGal80 (Gal80 under a tubulin promoter), which can suppress the transgene expression. Application of a heat shock triggers mitotic recombination in heterozygous cells at the FRT sites, leading to the generation of two identifiable clones (Figure 1A). This results in one clone with two copies of ubi-GFP (GFP-positive) and another with two copies of TubGal80 (GFP-negative). Both clones are easily identifiable against the background, which weakly expresses GFP in heterozygous cells (Figures 1B–1E).

We dissected eye-antennal imaginal discs from animals producing control clones without Aβ42 expression and examined their retinal neurons. The two sister clones produced in a control genetic background comprised GFP-positive and GFP-negative neuronal populations (Figure 1D). For the purposes of comparison, we will consider clones that do not produce Aβ42 in this context to be wild-type (WT). Clone sizes were quantified in ImageJ by drawing a region of interest around each clone and measuring the area of that region. In controls, we found no significant difference between GFP-positive WT and GFP-negative WT clones (Figure 1F, p = 0.63). The retinal neurons of these eye discs were normally arranged, as evident from Elav staining to mark the nuclei of retinal neurons (Robinow and White, 1991) (Figure 1D). The adult eyes were normal in appearance (Figure 1B).

To study the interactions between Aβ42-expressing and WT neurons, we generated GFP-positive clones that express human-Aβ42 under the retinal neuron driver GMR (Glass Multiple Repeat)-Gal4, whereas GFP-negative clones comprise WT neurons owing to the presence of two copies of the Gal4 repressor, TubGal80 (Figure 1C). GMR-Gal4 drives expression in the differentiating retinal neurons and not in the neuronal precursor cells, allowing us to model AD pathology early in the disease progression. Aβ42 expression in this system triggers formation of extracellular plaques, as previously described (Tare et al., 2011) (Figure S1). Because both clones originate from a single recombination event from one progenitor cell, we expected them to be equivalent in size. Instead, we found that the WT clones are significantly reduced in size compared with Aβ42-expressing sister clones (Figure 1F, ∗∗p < 0.01). Additionally, we observed spacing defects indicating the loss of retinal neurons from both Aβ42-expressing clones and WT sister clones (Figure 1E).

### Aβ42-Expressing Clones and Controls Show Similar Levels of Cell Proliferation

**Figure 2:**
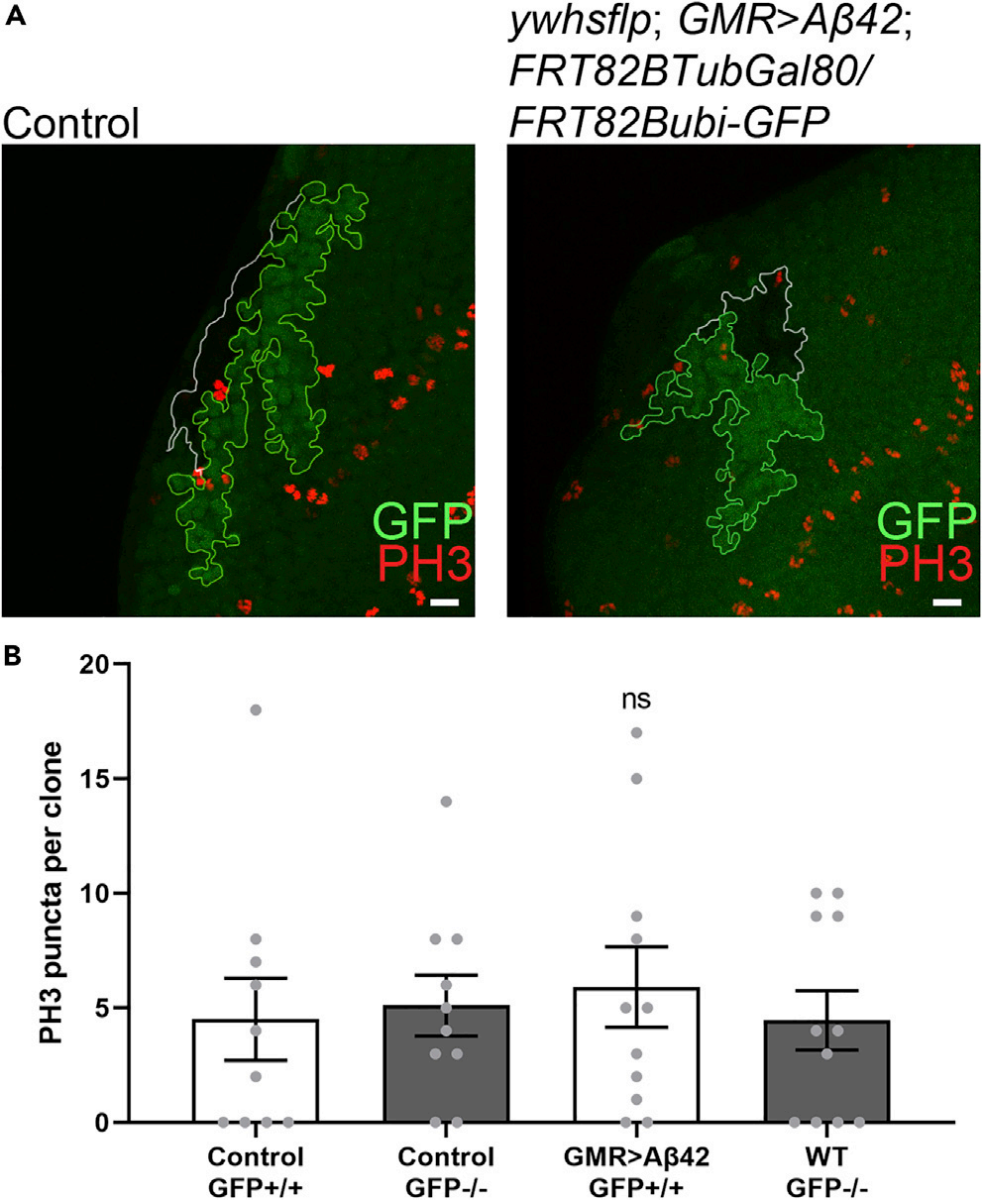
Cell Proliferation Is Similar Among Aβ42-Expressing Clones and Controls (A) Phosphohistone H3 (PH3) staining marks proliferating cells. Scale bars, 10 μm. (B) PH3 puncta were quantified for GFP-positive and GFP-negative WT control clones (N = 10) and Aβ42-expressing and WT clones (N = 11). Data are presented as mean ± SEM. Statistical analysis was done using one-way ANOVA with Tukey's post hoc to compare these four groups. No significant differences were observed. See also Table S2.

A difference in the size of clones could be due to either excessive proliferation or cell death in clonal cell populations. We stained with an antibody against phospho-histone H3 (PH3), a sensitive and reliable marker of mitosis (Hendzel et al., 1997) (Figure 2A). The number of PH3-positive puncta was found to be comparable among all four groups, GFP-positive and GFP-negative sister clone controls, and GFP-positive Aβ42-expressing and GFP-negative WT sister clones (Figure 2B, see Table S2 for raw values). These results suggested that the difference in size between Aβ42-expressing clones and their WT sister clones could be due to cell death rather than changes in proliferation.

### Expression of Aβ42 in Clones Triggers Cell Death in Nearby WT Cells

**Figure 3:**
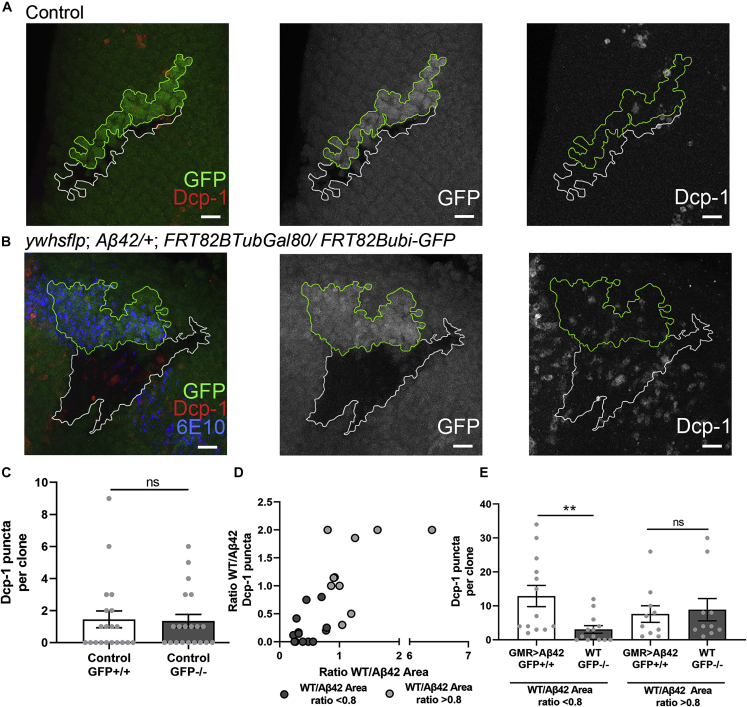
Aβ42-Expressing Clones Trigger Cell Death in Surrounding WT Cells (A) Control eye discs were stained with Dcp-1 to mark cell death. Scale bars, 10 μm. (B) Eye discs with Aβ42-expressing and WT clones stained with Dcp-1. Scale bars, 10 μm. (C) Cell death was compared in control clones. Data are presented as mean ± SEM. Statistical analysis was done using two-way unpaired Student's t test (N = 20, p = 0.88). (D) For each pair of WT and Aβ42-expressing clones, a ratio of WT/Aβ42 clone area and WT/Aβ42 Dcp-1 puncta number was calculated and plotted. (E) WT and Aβ42-expressing clones were divided into subgroup based on the ratio of WT/Aβ42-expressing clone area. Data are presented as mean ± SEM. In clone pairs in which WT clone size is substantially decreased, WT clones show less cell death (N = 13, ∗∗p < 0.01, two-way unpaired Student's t test). WT clones comparable in size with Aβ42-expressing sister clones show similar levels of cell death to Aβ42-expressing clones (N = 10, p = 0.75, two-way unpaired Student's t test). See also Table S2.

To quantify cell death, we stained eye imaginal discs with an antibody against activated caspase, Dcp-1 (Song et al., 1997). In the control eye discs, GFP-positive and GFP-negative WT clones showed a low level of cell death, averaging only ~1.5 puncta per clone (Figures 3A and 3C). However, a higher level of cell death was observed in WT sister clones of Aβ42-expressing clones (Figure 3B). To understand the relationship between clone size and cell death, we calculated the ratio of the clone area of WT to Aβ42-expressing clone and the ratio of cell death in WT to Aβ42-expressing clone (Figure 3D). The rationale was that WT cells start dying as soon as clones are generated and Aβ42 expression begins, and as a result, they are smaller in size when eye discs are stained. Of these WT clones, a subset was comparable in size with their Aβ42-expressing sister clones (WT/Aβ42 clone area >0.8). These WT sister clones showed a substantial level of cell death (see Table S2 for raw values). By contrast, those WT clones that were highly reduced in size compared with Aβ42-expressing sister clones showed low levels of cell death.

We reasoned that the difference between these two groups may reflect the progression of the pathology along the developmental time window in this model. We compared the cell death data for these two groups (Figure 3E). Our results suggest that initially, WT and Aβ42-expressing clones grow at equivalent rates after arising from one single progenitor cell until the presence of Aβ42-expressing cells begins to trigger cell death in the WT clones. WT cells die, leading to a decrease in the size of WT clones. This suggests that changes in cell signaling, downstream of Aβ42 accumulation, could result in the sensitization of WT cells to pathological signals emanating from nearby Aβ42-expressing cells, resulting in decreased fitness and death of WT cells.

### Modulation of the JNK Signaling Pathway in Aβ42-Expressing Clones Causes Cell Death in WT Cells

**Figure 4:**
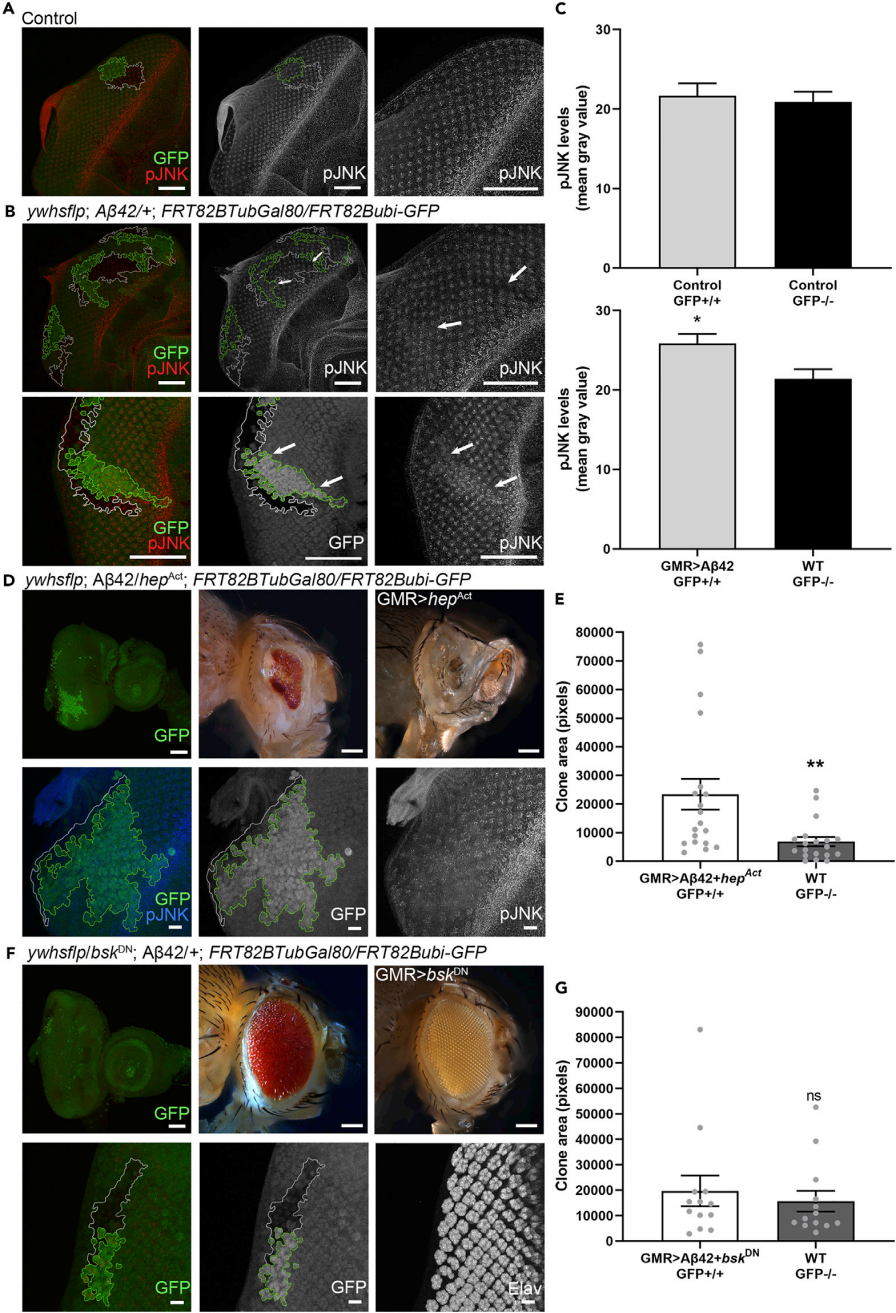
Modulation of the JNK Signaling Pathway in Aβ42-Expressing Clones Dictates the Survival or Elimination of WT Cells (A) Control clones show a regular pattern of pJNK staining throughout the eye disc. This pattern is consistent across WT GFP-positive and WT GFP-negative clones. Scale bars, 50 μm. (B) Eye disc with GFP-positive clones expressing Aβ42. pJNK levels are increased in Aβ42-expressing clones. Magnification shows increased pJNK signal overlapping with the Aβ42-expressing clone. The upregulation of pJNK expression in Aβ42-expressing clones extends into WT sister clones. Scale bars, 50 μm. (C) pJNK levels were compared for control sister clones and Aβ42-expressing and WT sister clones. No significant difference was observed between control clones (N = 17, p = 0.72, two-way unpaired Student's t test). Data are presented as mean ± SEM. We observed an increase in pJNK mean gray value in Aβ42-expressing clones (N = 17, ∗*p* < 0.05, two-way unpaired Student's t test). Because pJNK staining results in strong signal in the optic stalk, for pJNK mean gray value calculation, we selected for analysis the region of the z stack that did not substantially overlap with the optic stalk staining. (D) To test the effects of upregulating JNK signaling, we expressed *hep*^*Act*^ in Aβ42-expressing cells. Expression of *hep*^*Act*^ in clones led to a non-autonomous increase in cell death throughout the entire eye, resulting in highly reduced eyes. Expression of *hep*^*Act*^ in all retinal neurons using GMR-Gal4 (GMR > *hep*^*Act*^) triggers neurodegeneration. WT sister clones are highly reduced in size compared with clones expressing Aβ42 and *hep*^*Act*^. Staining for pJNK revealed a region of increased pJNK levels overlapping with the area of the GFP-positive clone. (E) GFP-positive clones expressing Aβ42 and *hep*^*Act*^ and GFP-negative WT clone sizes were quantified. WT sister clones are significantly reduced in size compared with clones expressing Aβ42 and *hep*^*Act*^ (N = 19, ∗∗p < 0.01, two-tailed unpaired Student's t test). Data are presented as mean ± SEM. (F) To test the effects of downregulating JNK activity, we expressed *bsk*^DN^ in Aβ42-expressing neurons. Adult flies expressing *bsk*^DN^ in Aβ42-expressing neuronal clones show approximately WT eye size. Expression of *bsk*^DN^ in all retinal neurons using GMR-Gal4 (GMR > *bsk*^DN^) results in eyes that are normal in appearance (*ywhsflp*/bsk^DN^; Aβ42/+; FRT82BTubGal80/FRT82ubi-GFP). Expression of *bsk*^DN^ in Aβ42-expressing cells rescues the size of WT clones. Staining retinal neurons with Elav shows irregular spacing in the Aβ42-expressing clone but normal spacing in the WT clone. (G) Areas of GFP-positive clones expressing Aβ42 and *bsk*^DN^ were compared with GFP-negative WT sister clones, showing no significant difference in size (N = 13, p = 0.58, two-tailed unpaired Student's t test). Data are presented as mean ± SEM. See also Figures S1 and S2 and Table S1.

Previous research in flies and humans has implicated activation of the highly conserved JNK signaling pathway in AD (Irwin et al., 2020; Sarkar et al., 2018; Tare et al., 2011; Wang et al., 2014; Yarza et al., 2015). Phosphorylation of JNK transcriptionally activates apoptosis, and increased levels of pJNK have been reported in patients with AD (Wang et al., 2014). We stained with an antibody specific to activated, phosphorylated JNK (pJNK). pJNK produces a regular pattern of staining across eye discs (Figure 4A). In control animals, the pattern of pJNK expression remained consistent across GFP-positive WT clones and GFP-negative WT clones (Figure 4A). Aβ42-expressing clones showed subtle increases in pJNK signal, comprising a haze seen over the regular pattern of staining in controls (Figure 4B). Furthermore, we observed local regions of increased pJNK within Aβ42-expressing clones, which appeared to extend into the area of WT clones (Figure 4B). Quantification showed a modest but significant increase in pJNK levels in Aβ42-expressing clones compared with WT clones (Figure 4C).

**Figure 5:**
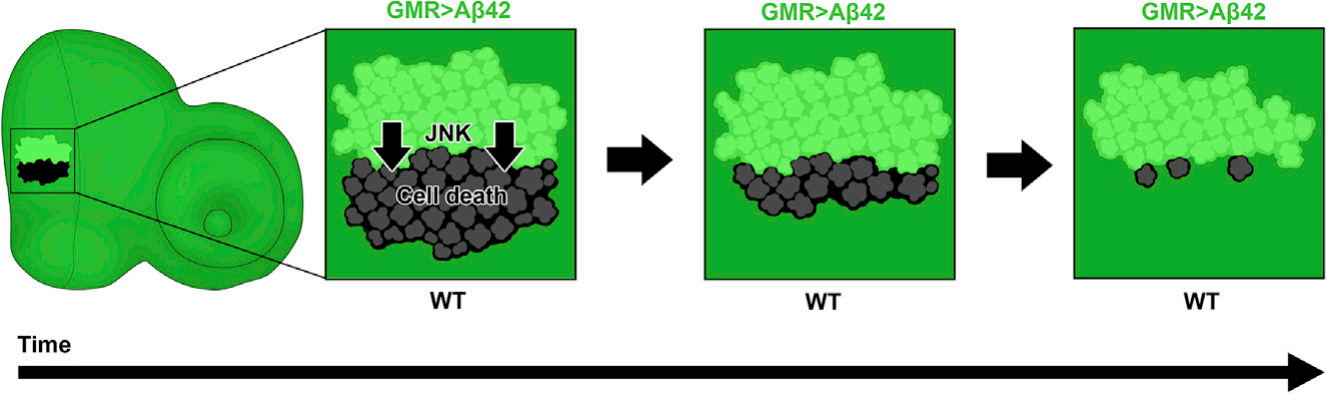
Signaling Cross Talk Between Aβ42-Expressing and WT Cells Results in Death of WT Cells Activation of JNK in Aβ42-expressing clones triggers preferential cell death in WT clones, leading to a decrease in overall WT clone size over time. Although cell death occurs in Aβ42-expressing clones, WT clones are affected first. Aβ42-expressing clones with few or no remaining WT cells could also be observed.

We then introduced genetic manipulations of the core JNK pathway components in Aβ42-expressing clones. We expressed a constitutively active form of JNK kinase homolog *hemipterous, hep*^*Act*^ (Glise et al., 1995; Tournier et al., 1997), resulting in a significant reduction in the size of WT sister clones (Figures 4D, 4E; ∗∗p < 0.01). Staining with pJNK revealed an increase in pJNK signal over the Aβ42 and *hep*^*Act*^ expressing clone (Figure 4D). Since JNK is ubiquitously expressed, we could see changes in levels of JNK signals. Interestingly, when we examined adult flies with *hep*^*Act*^ expression in Aβ42-expressing clones, we saw a strong reduction in the size of the entire eye (Figure 4D). These results provide additional evidence that Aβ42-expressing cells signal to surrounding tissue, resulting in the loss of WT cells, and that the resultant cell death is mediated at least in part by the JNK pathway. Finally, we decreased JNK activity by expressing a dominant negative form of *Drosophila* JNK homolog, *basket* (*bsk*) (Sluss et al., 1996) (Figure 4F). Interestingly, expressing *bsk*^DN^ in Aβ42-expressing clones restored the size of the WT sister clones (Figure 4F). We saw no significant difference in size between GFP-positive and GFP-negative clones (Figure 4G; p = 0.58). We further verified the presence of Aβ42 plaques in clones expressing both Aβ42 and *bsk*^DN^ by staining (Figure S1). We found robust induction of Aβ42 expression in the background where *bsk*^DN^ levels are also upregulated. Despite the robust accumulation of Aβ42, there is a significant rescue in the size of WT sister clones when *bsk*^DN^ is expressed. Overall, these results demonstrate that activation of the JNK pathway in Aβ42-expressing clones triggers cell death in WT sister clones (Figure 5).

## Discussion

It has been debated for a long time about which cellular population is killed to exhibit progressive neurodegenerative phenotypes in transgenic gain-of-function models of Aβ42 expression, since in these experimental models all the cells within a field are expressing high levels of Aβ42. Therefore, the experimental models using uniform ectopic expression of Aβ42 recapitulate the cell death dynamics of AD at a local level. In order to generate insights into the mechanism we developed a two-clone system. This two-clone approach takes advantage of genetic tools in *Drosophila* to uncover new insights into the interactions between Aβ42-expressing and WT neurons to model aspects of AD pathology. The utility of this system is that we can generate two labeled populations of clones from the same progenitor cell and express Aβ42 in one population of differentiating retinal neurons, which facilitates the study of pathways implicated in AD pathology. We show evidence that Aβ42-expressing cells produce aberrant signals that instead sensitize their WT neighbors to cell death (Figure 1).

Previous research has established that transgenic expression of Aβ42 throughout the brain or retina in *Drosophila* results in increased cell death and neurodegeneration (Cao et al., 2008; Moran et al., 2013; Sarkar et al., 2018; Tare et al., 2011). We see here that there is a more complicated cross talk that preferentially impacts WT cells before Aβ42-expressing cells. A widespread cell death is not a feature of normal, healthy aging, and previous research has suggested that aberrant changes to cell signaling downstream of Aβ42 accumulation lead to progressive neurodegeneration (Deshpande et al., 2019; Gogia et al., 2021; Sarkar et al., 2016; Yeates et al., 2019). Here we show evidence that Aβ42-expression triggers an increase in JNK activation that ultimately leads to the death of neighboring WT cells in the sister clone (Figure 5).

The JNK pathway regulates cell death through transcriptional activation of pro-apoptotic factors. The subtle increase in pJNK signal we observed was consistent with previous research showing an increase in pJNK in the eye discs of flies expressing Aβ42 (Sarkar et al., 2018; Tare et al., 2011). Importantly, our genetic manipulations of the JNK pathway then supported the idea that JNK signals may be emanating from Aβ42-expressing cells. Expression of *hep*^*Act*^ in Aβ42-expressing clones resulted in a substantial decrease in WT clone size. Previous research has shown that *hep*^*Act*^ expression alone activates the JNK pathway, resulting in cell death. Expression of both *hep*^*Act*^ and Aβ42 together worsens neurodegeneration (Sarkar et al., 2018; Tare et al., 2011). Finally, expression of *bsk*^DN^ in Aβ42-expressing clones restored the size of the WT sister clones to normal. Expression of *bsk*^DN^ in Aβ42-expressing clones appears to rescue WT clone size without impacting Aβ42 plaque production. Expression of *bsk*^DN^ and Aβ42 in the entire GMR domain yields robust Aβ42 plaque formation, shown by staining. Similarly, clones expressing *bsk*^DN^ and Aβ42 show plaque formation (Figure S1), providing evidence against the possibility that *bsk*^DN^ expression is rescuing WT clone size simply by reducing the amount of Aβ42 overall. Blocking JNK signaling in Aβ42-expressing neurons restored the size of WT clones, strongly indicating that the JNK pathway plays a critical role in the cell death of WT cells in AD.

What remains unknown is the mechanism by which JNK activation in Aβ42-expressing clones could be impacting the survival of WT clones. One possibility could be signaling interactions mediated through vesicular transport. Because the death of WT cells suggested a signal coming from Aβ42-expressing cells, we introduced mutations impairing cellular transport in Aβ42-expressing clones by expressing dominant negative Rab5^DN^ (Wucherpfennig et al., 2003) or *Drosophila* dynamin ortholog Shibire^DN^ (Moline et al., 1999) (Figure S2). However, by blocking all cellular transport, including potentially both cues for cell survival as well as cell death, these manipulations failed to rescue WT clone size (Figure S2). Further research will be needed to understand what aspects of cell-cell communication are altered between Aβ42-expressing and WT cells. One possibility is that cell competition could play a role in this process. Isoforms of the cell membrane protein Flower encode cell fitness relative to neighboring cells, with less fit cells being targeted for apoptosis (Coelho and Moreno, 2019; Coelho et al., 2018).

The principles of the two-clone system can also be applied to study the interactions between tau-expressing and WT sister clones, as well as clones expressing both Aβ42 and tau. This will be important to explore especially as JNK has been implicated in tau hyperphosphorylation (Ferrer et al., 2001; Ma et al., 2009; Ploia et al., 2011). This system provides a useful blueprint to study cross talk between cell populations in neurodegenerative disease and potentially identify new biomarkers differentially regulated between Aβ42-expressing and WT neurons. Better understanding of the local context of cell death in progressive neurodegenerative disease is a vital next step in developing new interventions to slow or halt disease progression.

### Limitations of the Study

Aβ42 accumulation is one component of AD, and further research in this system is necessary to examine the role of tau as well as the combination of Aβ42 and tau in clones. Furthermore, the GMR-Gal4 driver drives expression in the developing retina, including retinal neurons and other cell types of the retina.

### Resource Availability

#### Lead Contact

Further information and requests for resources and reagents should be directed to and will be fulfilled by the Lead Contact, Amit Singh (asingh1@udayton.edu).

#### Materials Availability

All fly lines generated in this study are available from the Lead Contact upon reasonable request.

#### Data and Code Availability

We include the raw data used to generate the figures in Tables S1 and S2. Any other original source data for figures in the paper are available upon request to the Lead Contact.

## Methods

All methods can be found in the accompanying Transparent Methods supplemental file.

## Supplemental Information

Document S1. Transparent Methods, Figures S1 and S2, and Tables S1 and S2
