# Supplementary material for: A Two-Clone Approach to Study Signaling Interactions among Neuronal Cells in a Pre-clinical Alzheimer's Disease Model
Source: iScience. 2020 Nov 18;23(12):101823. doi: 10.1016/j.isci.2020.101823 (PMC7724150; doi:10.1016/j.isci.2020.101823)
Supplement: Document S1. Transparent Methods, Figures S1 and S2, and Tables S1 and S2 [file mmc1.pdf]

iScience, Volume *23*

Supplemental Information

A Two-Clone Approach to Study Signaling Interactions among Neuronal Cells in a Pre-clinical Alzheimer's Disease Model

Catherine J. Yeates, Ankita Sarkar, Prajakta Deshpande, Madhuri Kango-Singh, and Amit Singh

Supplemental Information

Condition	Mean Area (pixels)	SD	SEM	N
**Figure 1**				
Control GFP+/+	17951	11401	2082	30
Control GFP-/-	14547	9430	1722	30
GMR>Aβ42 GFP+/+	22259	13665	2379	33
WT GFP-/-	12140	8885	1547	33
**Figure 4**				
GMR>Aβ42+*hep*^{Act} GFP+/+	23378	23505	5392	19
WT GFP-/-	6793	6976	1600	19
GMR>Aβ42+*bsk*^{DN} GFP+/+	19708	21781	6041	13
WT GFP-/-	15639	14762	4094	13
**Figure S1**				
GMR>Aβ42+*Rab5*^{DN} GFP+/+	16053	12464	2787	20
WT GFP-/-	9928	5325	1191	20
GMR>Aβ42+*shi*^{DN} GFP+/+	20849	11820	2712	19
WT GFP-/-	7860	5995	1375	19

Table S1. Clone area raw data. Related to Figures 1, 4, and S1.

Condition	Mean PH3 Puncta	SD	SEM	N
**Figure 2**				
Control GFP+/+	4.5	5.7	1.8	10
Control GFP-/-	5.1	4.2	1.3	10
GMR>Aβ42 GFP+/+	5.9	5.8	1.8	11
WT GFP-/-	4.5	4.3	1.3	11
**Condition**	**Mean Dcp-1 Puncta**	**SD**	**SEM**	**N**
**Figure 3**				
Control GFP+/+	1.5	2.4	0.5	20
Control GFP-/-	1.4	1.8	0.4	20
GMR>Aβ42 GFP+/+ Total	10.6	10.1	2.1	23
WT GFP-/- Total	5.6	7.8	1.6	23
GMR>Aβ42 GFP+/+ WT/Aβ42 ratio <0.8	12.9	11.3	3.1	13
WT GFP-/- WT/Aβ42 ratio <0.8	3.1	4.0	1.1	13
GMR>Aβ42 GFP+/+ WT/Aβ42 ratio >0.8	7.6	7.7	2.4	10
WT GFP-/- WT/Aβ42 ratio >0.8	8.9	10.4	3.3	10

Table S2. Raw data of PH3 and Dcp-1 puncta. Related to Figures 2 and 3.

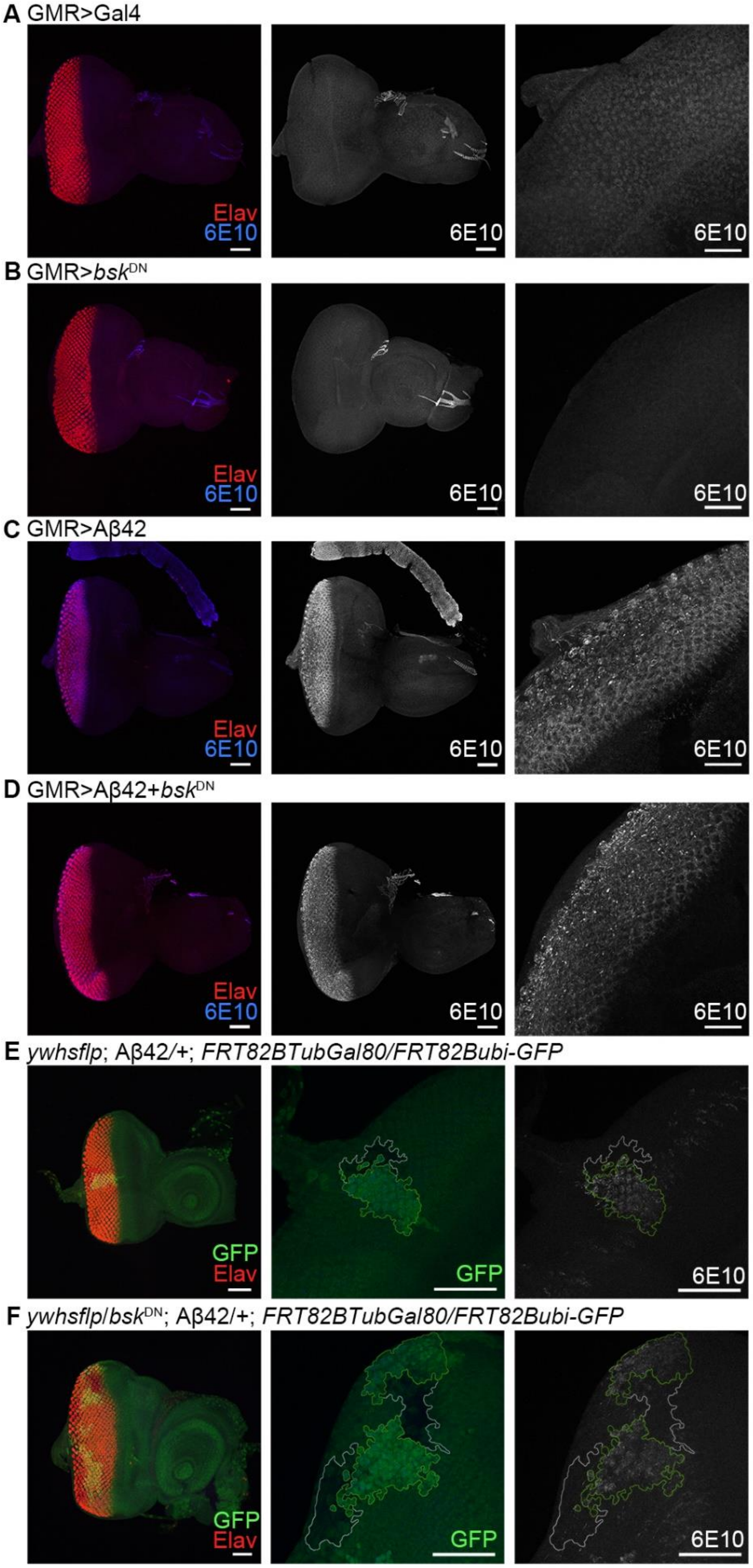
A GMR>Gal4
Elav
6E10
6E10
6E10
B GMR>bsk^DN
Elav
6E10
6E10
6E10
C GMR>Aβ42
Elav
6E10
6E10
6E10
D GMR>Aβ42+bsk^DN
Elav
6E10
6E10
6E10
E ywhsflp; Aβ42/+; FRT82BTubGal80/FRT82Bubi-GFP
GFP
Elav
GFP
6E10
F ywhsflp/bsk^DN; Aβ42/+; FRT82BTubGal80/FRT82Bubi-GFP
GFP
Elav
GFP
6E10

Figure S1. Aβ42 plaque accumulation occurs in the presence of *bsk*^{DN}. Related to Figure 4.

(A), Eye discs were stained with 6E10 to mark Aβ42. GMR-Gal4 driver controls as well as (B) eye discs expressing *bsk*^{DN} throughout the GMR domain show normally arranged retinal neurons (Elav staining) and no Aβ42, as expected. Scale bars for 20x images of entire eye discs, 50µm. Scale bars for higher magnification view, 50µm. (C), Expression of Aβ42 in the entire eye using GMR-Gal4 results in plaque accumulation and strong 6E10 staining. Scale bars as in (A) and (B). (D), Expression of Aβ42+*bsk*^{DN} results in Aβ42 plaque accumulation. Scale bars as in (A) and (B). (E), Expression of Aβ42 using the two-clone system results in 6E10 signal in the GFP-positive clone. Scale bars for 20x images of entire eye discs, 50µm. Scale bars for higher magnification view, 50µm. (F), Expression of Aβ42+*bsk*^{DN} similarly results in a 6E10 signal in the GFP-positive clones. Expression of *bsk*^{DN} does not appear to interfere with the accumulation of Aβ42 plaques. This suggests that the rescue of WT sister clones in this condition occurs even in the presence of robust Aβ42 plaque accumulation in Aβ42+*bsk*^{DN} clones. Scale bars as in (E).

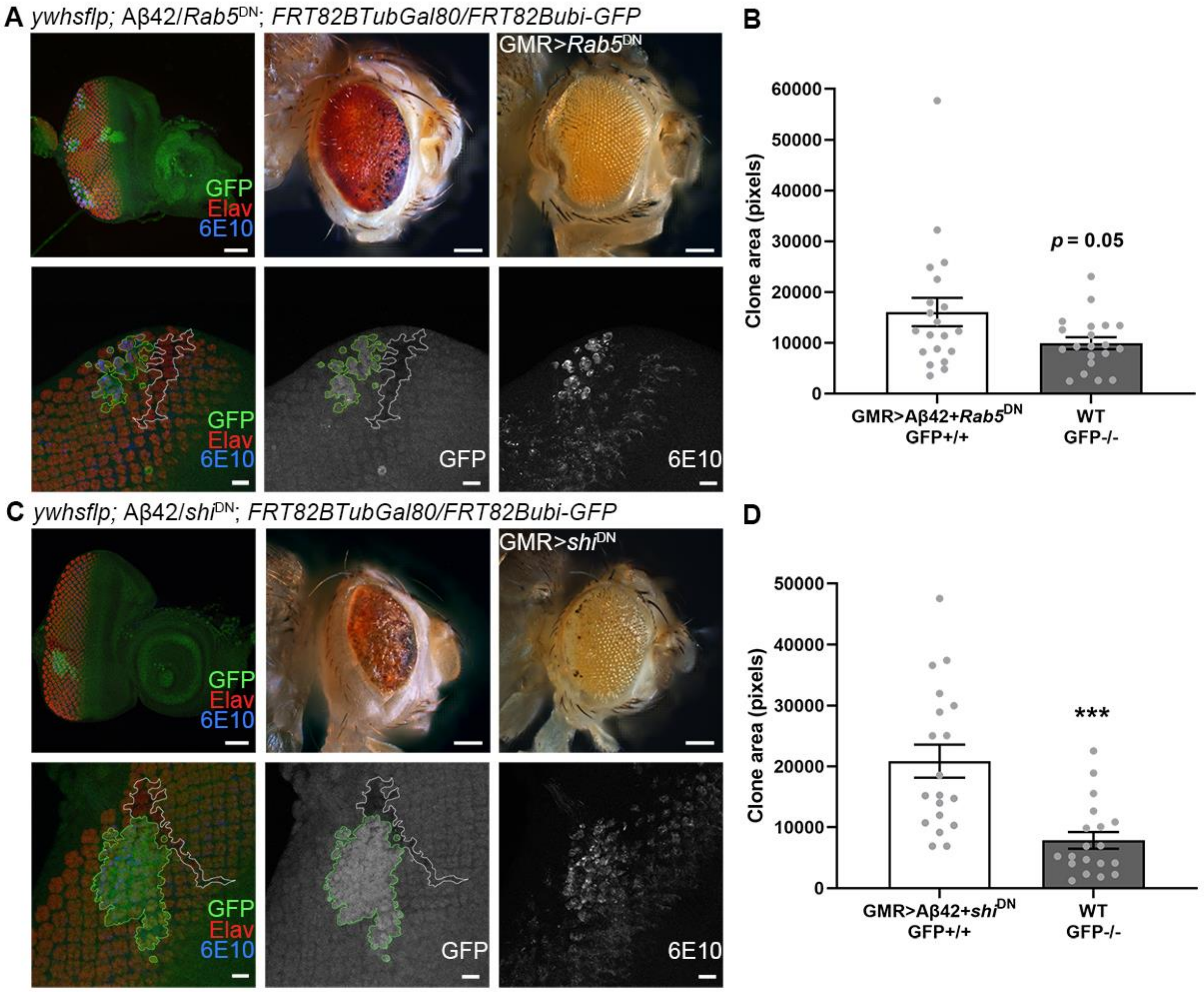

Figure S2. Expression of *Rab5*^{DN} or *shi*^{DN} in Aβ42-expressing clones failed to rescue WT clone size. Related to Figure 4.

(A), Expression of *Rab5*^{DN} in Aβ42-expressing clones results in a partial rescue of clone size. Scale bar for 20x image of clones, 50µm. Adult flies of this genotype show slight roughness and necrotic spots. When *Rab5*^{DN} is expressed in all retinal neurons using GMR-Gal4 (GMR>*Rab5*^{DN}), the eye is grossly normal with a slight malformation of eye shape on the posterior side. Scale bar, 100µm. In animals expressing *Rab5*^{DN} in Aβ42-expressing clones, sizes of WT clones show an intermediate phenotype. 6E10 staining marks the Aβ42 plaques. *Rab5*^{DN} expression led to a block in cell transport as seen by Aβ42 remaining trapped inside cells rather than being exported out. Scale bars for 40x images, 10µm. (B), Graph showing quantification of clone size comparing Aβ42+*Rab5*^{DN} expressing clones to WT sister clones (two-tailed, unpaired Student's *t* test, N = 20; $p = 0.05$.). (C), Eye disc with *shi*^{DN} expressed in Aβ42-expressing clones. Scale bar for 20x image of clones, 50µm. Adult flies expressing *shi*^{DN} in clones show overall reduction in eye size as well as dark spots along the anterior margin. *shi*^{DN} expression in all retinal neurons by GMR-Gal4 (GMR>*shi*^{DN}) results in mild aberrations. Scale bars, 100µm. *shi*^{DN} expression in Aβ42-expressing clones failed to rescue the size of WT clones. Scale bars for 40x images, 10µm. (D), Sizes of GFP-positive clones expressing Aβ42 and *shi*^{DN} and GFP-negative WT sister clones were compared using Student's *t* test (N = 19). WT sister clones are significantly reduced in size (***$p <$ 0.001).

Transparent Methods

Experimental Model and Subject Details

Fly stocks used in this study are described on FlyBase (https://flybase.org). The Gal4/UAS system was used for targeted misexpression studies (Brand and Perrimon, 1993). The Glass Multiple Repeat driver line (GMR-Gal4) was used to drive expression of Aβ42 in the developing retina (Moses and Rubin, 1991). A UAS-Aβ42 line with two tandem copies of human Aβ42 fused to a signal peptide was used (Casas-Tinto et al., 2011). This line was recombined with the retinal neuron driver, GMR-Gal4 (GMR-Gal4>UAS-Aβ42, abbreviated simply as Aβ42) (Tare et al., 2011). Other stocks used in this study include UAS-*bsk*^{DN} (Adachi-Yamada et al., 1999), UAS-*hep*^{Act} (Weber et al., 2000), and UAS-*Rab5*^{DN} (Zhang et al., 2007).

Clonal Analysis

The FLP/FRT system was used to generate clones through mitotic recombination. A Flippase (FLP) mediates recombination at Flippase Recognition Targets (FRT). We used transgenic lines in which FRT sites were inserted ahead of the sequence driving expression of either Gal80 under a tubulin promoter (FRT82BTubGal80) or GFP under a ubiquitin promoter (FRT82Bubi-GFP) on the third chromosome. We generated heterozygous progeny of the genotype *y w hsflp; GMR-Gal4>UAS-Aβ42/+; FRT82BTubGal80/FRT82Bubi-GFP*.

Parental lines of the cross producing these larvae were allowed to lay eggs for 8 hours at 25°C. We applied a heat shock 24 hours from the midpoint of the egg lay period to trigger mitotic recombination at the first instar larval stage. The 60-minute heat shock at 38°C was applied by placing vials in a water bath. Heat shocked vials were then transferred to 29°C until the animals reached the wandering third instar larval stage, at which point eye-antennal imaginal discs were dissected. The heat shock triggers mitotic recombination at the FRT points, resulting in two populations of cells: sister clones with either ubi-GFP/ubi-GFP or TubGal80/TubGal80. In TubGal80/TubGal80 clones, the Gal4 repressor Gal80 is constitutively produced under a Tubulin promotor, blocking expression of Aβ42. GFP under a ubiquitin promoter marks tissue expressing Aβ42 in this background. These clones and those WT clones without Aβ42 transgene expression can be readily distinguished from each other and the background, which shows weak GFP expression.

Quantification of clone size

Analysis was done using the FIJI package of ImageJ. GFP-positive and GFP-negative clones were analyzed by defining regions of interest in ImageJ using the freehand selection tool and a Wacom pen tablet.

Statistical analysis

Statistical analysis was conducted in Graphpad Prism 8. Unpaired, two-tailed Student's *t*-tests were used to compare one experimental data set with one control data set, while one-way ANOVA with Tukey's post hoc test was used to compare multiple data sets. *p*-values are noted as follows: $*p < 0.05$; $**p < 0.01$; and $***p < 0.001$, ns; not significant.

Immunohistochemistry

Eye-antennal imaginal discs were dissected from wandering third instar larvae and fixed for 20 minutes in 4% paraformaldehyde (Oros et al., 2010; Singh et al., 2004; Singh et al., 2011). Fixation was followed by three ten-minute washes in 1x PBST, after which the tissue was stained overnight at 4°C in primary antibody. The following primary antibodies were used: rat anti-Elav (proneural marker which stains the nuclei of retinal neurons; 1:100, DSHB) (Robinow and White, 1991), mouse anti-6E10 (1:100, Covance), rabbit anti-pJNK (1:250, Cell Signaling), mouse anti-PH3 (1:250, Cell Signaling), and rabbit anti-Dcp-1 Asp216 (1:150, Cell Signaling) (Song et al., 1997). Eye-antennal imaginal discs were washed three times in 1x PBST and incubated in secondary antibody in the dark at room temperature for two hours. Secondary antibodies (Jackson Laboratories) used were goat anti-rat IgG conjugated with Cy5 (1:250), donkey anti-mouse IgG conjugated with Cy3 (1:300), anti-Mouse IgG conjugated with Cy5 (1:300), and donkey anti-rabbit IgG conjugated with Cy3 (1:250). After a final set of three washes in 1x PBST, discs

were mounted in Vectashield (Vector Laboratories). Eye discs were imaged using an Olympus Fluoview 3000 Confocal Microscope. Final images and figures were prepared using Adobe Photoshop Creative Cloud.

Adult eye imaging

Adult flies were frozen at -20°C for 2-4 hours. After the removal of its wings and legs, each fly was mounted on a needle in a horizontal orientation. Images were taken using a Zeiss ApoTome with AxioCam MRc5 and AxioVision software. Z-stacks were taken and merged to form the final image (Sarkar et al., 2018; Singh et al., 2019; Wittkorn et al., 2015). Images were then prepared using Adobe Photoshop Creative Cloud.

Supplemental References

Oros, S.M., Tare, M., Kango-Singh, M., and Singh, A. (2010). Dorsal eye selector pannier (pnr) suppresses the eye fate to define dorsal margin of the Drosophila eye. Dev Biol *346*, 258-271.
Sarkar, A., Gogia, N., Farley, K., Payton, L., and Singh, A. (2018). Characterization of a morphogenetic furrow specific Gal4 driver in the developing Drosophila eye. PLoS One *13*, e0196365.
Singh, A., Gogia, N., Chang, C.Y., and Sun, Y.H. (2019). Proximal fate marker homothorax marks the lateral extension of stalk-eyed fly Cyrtodopsis whitei. Genesis *57*, e23309.
Singh, A., Kango-Singh, M., Choi, K.W., and Sun, Y.H. (2004). Dorso-ventral asymmetric functions of teashirt in Drosophila eye development depend on spatial cues provided by early DV patterning genes. Mechanisms of development *121*, 365-370.
Singh, A., Tare, M., Kango-Singh, M., Son, W.S., Cho, K.O., and Choi, K.W. (2011). Opposing interactions between homothorax and Lobe define the ventral eye margin of Drosophila eye. Dev Biol *359*, 199-208.
Weber, U., Paricio, N., and Mlodzik, M. (2000). Jun mediates Frizzled-induced R3/R4 cell fate distinction and planar polarity determination in the Drosophila eye. Development *127*, 3619-3629.
Wittkorn, E., Sarkar, A., Garcia, K., Kango-Singh, M., and Singh, A. (2015). The Hippo pathway effector Yki downregulates Wg signaling to promote retinal differentiation in the Drosophila eye. Development *142*, 2002-2013.
Zhang, J., Schulze, K.L., Hiesinger, P.R., Suyama, K., Wang, S., Fish, M., Acar, M., Hoskins, R.A., Bellen, H.J., and Scott, M.P. (2007). Thirty-one flavors of Drosophila rab proteins. Genetics *176*, 1307-1322.
